# Sirt6-induced autophagy restricted TREM-1-mediated pyroptosis in ox-LDL-treated endothelial cells: relevance to prognostication of patients with acute myocardial infarction

**DOI:** 10.1038/s41420-019-0168-4

**Published:** 2019-04-12

**Authors:** Ye Zi, Yao Yi-An, Ji Bing, Lai Yan, Tong Jing, Guan Chun-Yu, Ping Fan, Lin Hao, Tang Jia-Ni, Hou Han-Jin, Chen Fei, Liu Xue-Bo

**Affiliations:** Department of Cardiology, Shanghai Tongji Hospital, Tongji University, Shanghai, China

## Abstract

Inflammation mediated by myeloid cells trigger receptors 1 (TREM-1) is important for atherosclerosis development, while sirtuin 6 (Sirt6) levels decrease in atheroscleoritc plaque. Here we demonstrate that oxidatively modified low density lipoprotein (ox-LDL)-treated endothelial cells (ECs) exhibited increased TREM-1-mediated pyroptosis and decreased Sirt6-induced autophagy. We show that high sTREM-1 and low sSirt6 levels were independent predictors of boosted endothelial microparticles (EMPs) on admission, and were associated with increased risk for all-cause mortality and major adverse cardiovascular events (MACE) at median 24 months (interquartile range, 18–26) follow-up in acute myocardial infarction (AMI) patients. Additionally, blockage of Sirt6-induced autophagy led to augmented TREM-1-mediated pyroptosis, whereas Sirt6 overexpression attenuated ECs inflammation and pyroptosis following ox-LDL treatment. Our findings indicate that TREM-1 and in a reversed trend Sirt6 appeared to be markers of endothelial inflammation with potential for use in risk stratification.

## Introduction

Inflammation plays a critical role in atherosclerosis and atherothrombosis beyond that of hypercholesteremia^1^. For instance, in the CANTOS trial, anti-inflammation treatment with canakinumab led to a significantly lower rate of recurrent cardiovascular events independent of lipid level^2^. Moreover, clinical and experimental data support an important role of endothelial inflammation in the genesis and progression of atherosclerosis^3^. We reported that biomarkers of inflammation such as interleukin (IL)-6, IL-1β, and high sensitivity C-reactive protein were associated with an increased risk of no ST-segment resolution and cardiovascular events in patients undergoing emergent percutaneous coronary intervention (PCI)^4^, and that level of endothelial microparticles (EMPs), which reflects endothelial damage, was increased in patients with acute coronary syndrome (ACS)^5^. Yet, to date, the mechanism underlying the association between increasing degree of endothelial inflammation in atherosclerosis and higher rates of cardiovascular events remains unclear.

In atherosclerotic lesions, especially in advanced plaques, there is increased cell death and inflammation, with the former augmenting the latter^6^. Cell death is traditionally ascribed to apoptosis and necrosis; however, other forms of cell death have been identified, including pyroptosis^7^. Pyroptosis is defined as inflammatory cell death, which is dependent on caspase-1 activation and pro-IL-1β, pro-IL-18 maturation^8^, and that is triggered by both infectious and noninfectious diseases. Recently, pyroptosis was associated with nicotine-promoted atherosclerosis^9^ and ox-LDL-induced macrophage death^10^, suggesting a critical role of pyroptosis in atherosclerosis and rendering its inhibition a therapeutic target. Autophagy, a “housekeeper” function for maintaining cellular homeostasis, may control cellular export and release of IL-1β^11^ and determine the modality of cell death progression, such as apoptosis, necrosis, and senescence^12^. Although inflammation might induce autophagy suppression and pyroptosis activation^13^, the relationship between pyroptosis and autophagy remains controversial.

The triggering receptor expressed on myeloid cells-1 (TREM-1), i.e., in neutrophils and monocytes/macrophages, is considered an amplifier of the innate immune response in infectious and noninfectious inflammation^14^. Recently, TREM-1 was shown to be a biomarker for atherosclerosis and ACS^4^, and evidence suggests that its inhibition using genetic or pharmacological methods may limit the development of experimental atherosclerosis^15^. TREM-1 also is expressed on vascular smooth muscle cells (VSMCs) and its activation promotes VSMCs inflammation, proliferation, and migration^16^. It remains unclear if TREM-1 is expressed on endothelial cells (ECs) and if it is associated with EC inflammation. Sirtuin 6 (Sirt6), a member of the evolutionarily conserved nicotinamide adenine dinucleotide-dependent histone deacetylases, has been found to be downregulated and to induce autophagy in diabetes mellitus and atherosclerotic plaque^17^. Sirt6 deficiency exacerbates endothelial senescence and chronic noninfective inflammation^18^. We hypothesized that Sirt6 downregulation may be a risk factor for poor prognosis and a trigger for upregulation of endothelial inflammation in ACS patients. The present study therefore was aimed at investigating whether TREM-1 upregulation and Sirt6 downregulation are associated with severe endothelial damage and poor prognosis in ACS patients, and to assess whether Sirt6-induced autophagy may restrict TREM-1-guided ECs pyroptosis following ox-LDL treatment.

## Results

### TREM-1-mediated endothelial cell pyroptosis following ox-LDL treatment

**Fig. 1:**
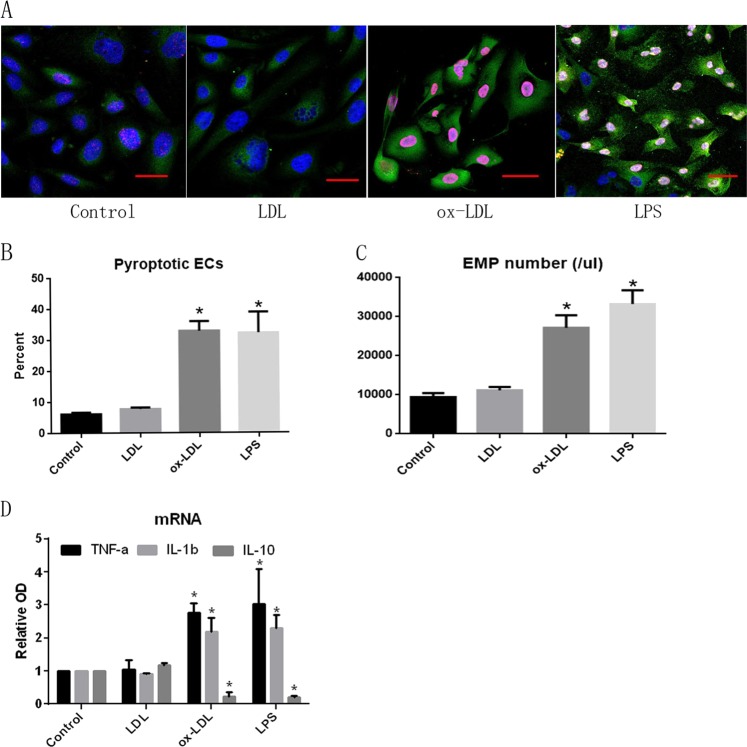
ox-LDL induces ECs pyroptosis. **a** Confocal detection of ox-LDL-induced pyroptotic cells formation. LDL was used as a negative control, and LPS as a positive control. Green: 488-labeled Casp1; red: TMR red-labeled pyroptotic nucleus; blue: DAPI-labeled natural nucleus; green and red double-labeled cells correspond to pyroptotic cells. *N* = 3. Scale bar: 50 μm. **b** Flow cytometry detecting ox-LDL-induced pyroptotic cells percentage. *N* = 3; *p < 0.05 versus LDL group. **c**. Flow cytometry detection of ox-LDL-induced EMPs number. *N* = 3; *p < 0.05 versus LDL group. **d** RT-PCR detection of mRNA expression of ox-LDL-induced inflammatory factors. *N* = 3; *p < 0.05 versus LDL group

As previously reported, we used ox-LDL (50 µg/ml) to induce EC damage; LDL (50 µg/ml) and LPS (1 µg/ml) were used in parallel as negative- and positive-control, respectively. As shown in Fig. 1a, three-color confocal imaging revealed that ox-LDL significantly induced EC pyroptosis. Two-color flow cytometry also showed that ox-LDL treatment induced a sixfold increase in pyroptotic EC number (Fig. 1b). EMP number increased following ox-LDL treatment (Fig. 1c). In terms of expression of inflammatory factors, as shown in Fig. 1d, TNF-α and IL-1β mRNA expression increased following ox-LDL treatment, whereas that of IL-10 decreased.

**Fig. 2:**
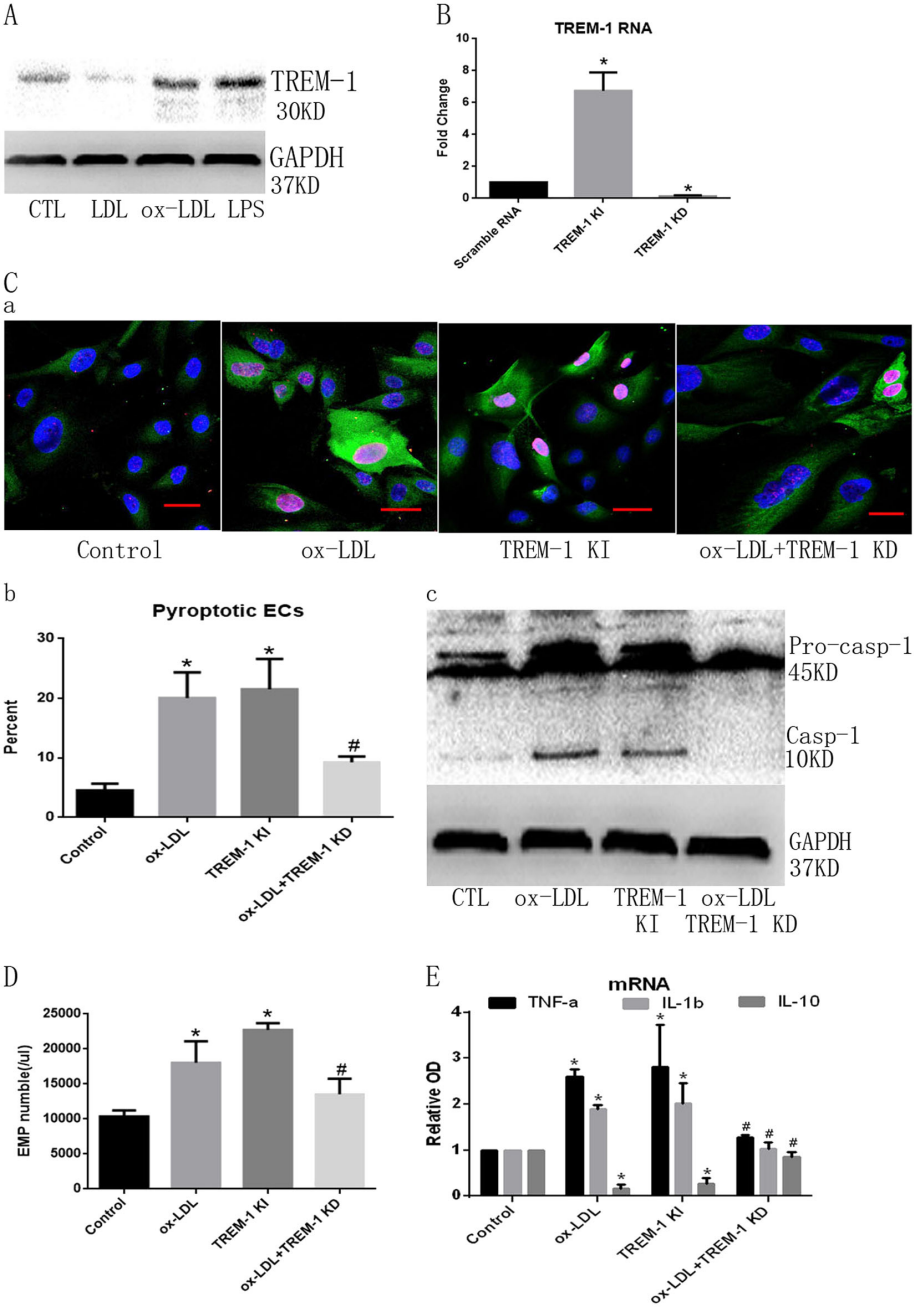
TREM-1 participates in ox-LDL-induced ECs pyroptosis. **a** ox-LDL and LPS-induced TREM-1 expression. **b** Adv TREM-1 or LV TREM-1 significantly changed TREM-1 mRNA expression. **c** TREM-1 KI or TREM-1 KD effectively regulated ECs pyroptosis. *N* = 3. *p < 0.05 versus control group; ^#^*p* < 0.05 versus ox-LDL group. TREM-1 regulated pyroptotic cells formation (**a**) (Scale bar: 50 μm) and cell percentage (**b**), and Casp1 maturation. **d** TREM-1 regulated EMPs release following ox-LDL treatment. *N* = 3. *p < 0.05 versus control group; ^#^*p* < 0.05 versus ox-LDL group. **e** TREM-1 regulated inflammatory factors mRNA expression following ox-LDL treatment. *N* = 3. *p < 0.05 versus control group; ^#^*p* < 0.05 versus ox-LDL group

To assess whether TREM-1 participates in ox-LDL-induced pyroptosis, we then examined TREM-1 expression in ECs. As shown in Fig. 2a, TREM-1 expression was much higher in ox-LDL or LPS group compared with LDL group, which indicated that TREM-1 may play a key role in ox-LDL-mediated EC pyroptosis. Then we used overexpression (TREM-1 KI, Adv TREM-1) or knockdown method (TREM-1 KD, LV TREM-1) to regulate EC TREM-1 expression, we found that adv TREM-1 or LV TREM-1 significantly changed TREM-1 mRNA expression (Fig. 2b). Interestingly, TREM-1 KI directly induced ECs pyroptosis and EMPs release, whereas TREM-1 KD nearly abolished ox-LDL-mediated ECs pyroptosis (Fig. 2c, d). Also, TREM-1 overexpression induced TNF-α and IL-1β mRNA expression, whereas it inhibited IL-10 expression. Additionally, TREM-1 KD nearly abolished the ox-LDL-mediated effects on inflammatory factors in ECs (Fig. 2e).

### ox-LDL restricted Sirt6-induced autophagy in ECs

**Fig. 3:**
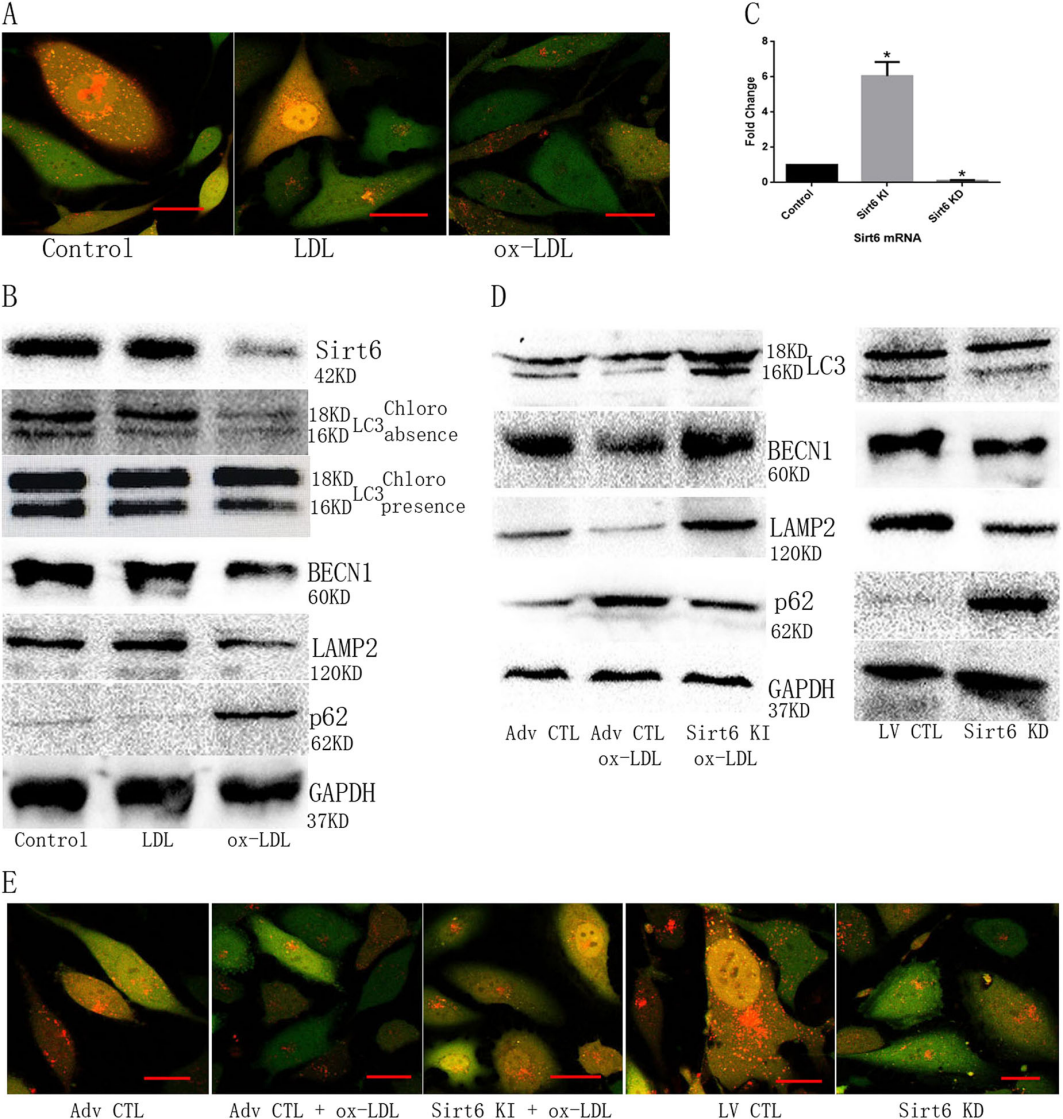
Sirt6 participates in ox-LDL-decreased ECs autophagy. **a** ox-LDL-decreased ECs autophagosome formation. Green, GFP; red, RFP-labeled LC3; yellow, green, and yellow double-labeled autophagosome. Scale bar: 100 μm. **b** ox-LDL inhibited Sirt6 expression and decreased autophagic biomarkers expression with or without chloroquine. Chloro, Chloroquine. **c** Adv Sirt6 or LV Sirt6 significantly changed Sirt6 mRNA expression. **d** Sirt6 KI or Sirt6 KD effectively regulated ECs autophagic biomarkers expression. **e** Sirt6 KI or Sirt6 KD effectively regulated ECs autophagosome formation. *N* = 3. Scale bar: 100 μm

To examine the change in Sirt6-induced autophagy in ECs under normal and ox-LDL conditions, we used mRFP-GFP-LC3 to stain for autophagosomes. As shown in Fig. 3a, ECs treated with ox-LDL contained less mRFP-GFP-LC3 vacuoles in the cytoplasm, indicating that ox-LDL inhibited EC autophagosome formation. Levels of the autophagic biomarkers decreased following ox-LDL treatment in the presence or absence of chloroquine (Fig. 3b). Interestingly, Sirt6 expression also was downregulated significantly in ECs treated with ox-LDL (Fig. 3b).

To determine the role of Sirt6 on ox-LDL-mediated EC autophagic inhibition, we established wide-type Sirt6 overexpression by means of recombinant adenovirus (Sirt6 KI, Adv Sirt6) and Sirt6 knockdown by means of recombinant lentivirus (Sirt6 KD, LV Sirt6), respectively (Fig. 3c). Based on western blot and mRFP-GFP-LC3 staining results, we found that Sirt6 KI rescued autophagic biomarkers expression and autophagosome formation in ox-LDL-treated ECs, whereas Sirt6 KD nearly abolished autophagy efflux in untreated ECs (Fig. 3d, e). The latter observations suggested that Sirt6 could relieve autophagy efflux in ox-LDL-treated ECs.

### TREM-1 mediated autophagy-inhibition in ox-LDL-treated ECs

**Fig. 4:**
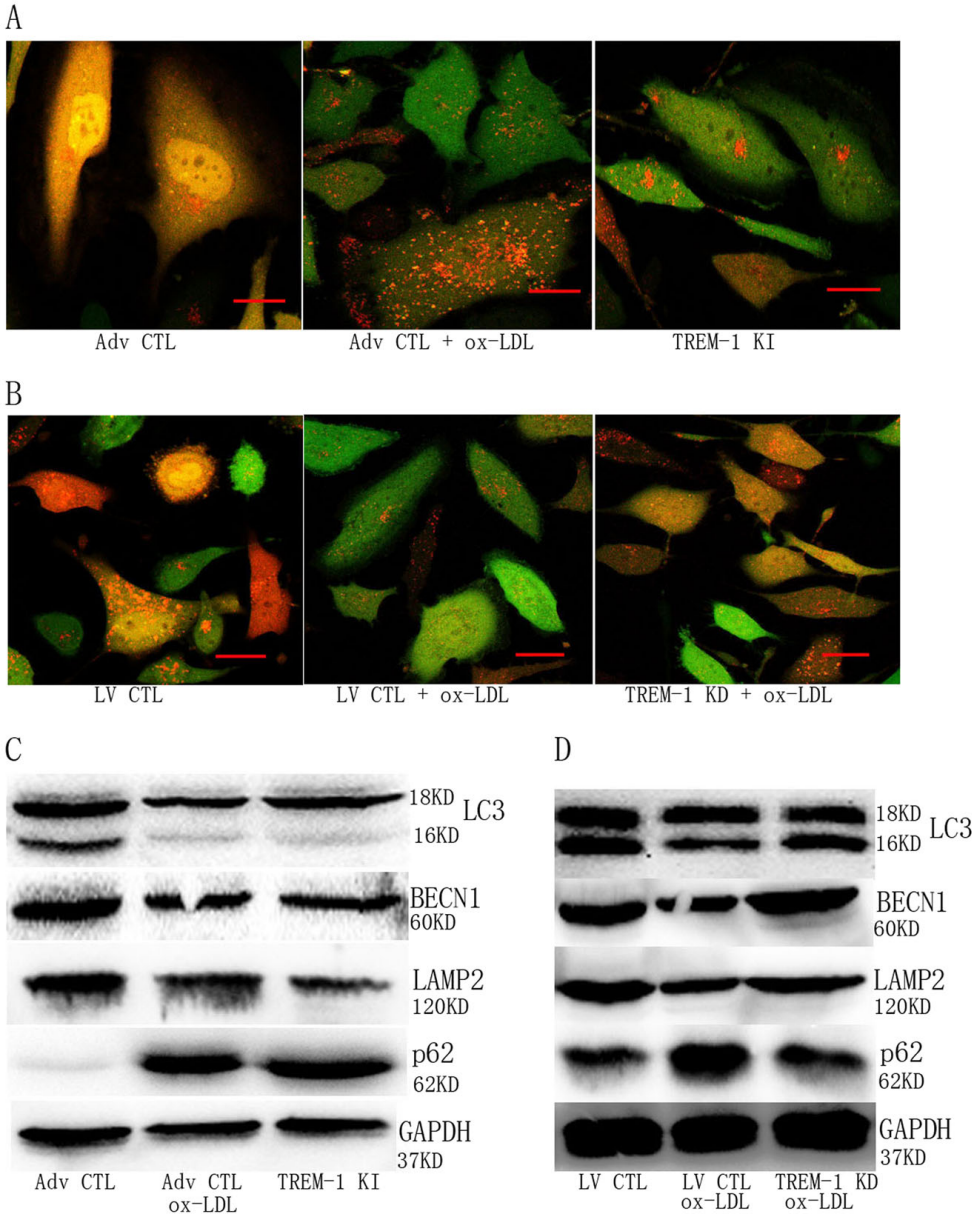
TREM-1 inhibits autophagy in ox-LDL-treated ECs. **a** TREM-1 KI directly inhibited autophagosome formation. Scale bar: 100 μm. **b** TREM-1 KD abrogated ox-LDL-mediated autophagosome formation inhibition. Scale bar: 100 μm. **c** TREM-1 KI directly inhibited autophagic biomarkers expression. **d** TREM-1 KD abrogated ox-LDL-mediated autophagic biomarkers expression inhibition. *N* = 3

To evaluate whether ox-LDL-induced autophagy inhibition is associated with TREM-1 activation, we directly regulated TREM-1 expression in ECs. As shown in Fig. 4a, b, TREM-1 KI directly impeded autophagosome formation as with ox-LDL treatment, whereas TREM-1 KD rescued autophagic efflux in ECs. Also, we detected autophagic biomarkers expression following TREM-1 regulation. Interestingly, TREM-1 KI decreased autophagic biomarkers expression in ECs (Fig. 4c). Moreover, TREM-1 KD induced autophagic biomarkers re-expression in ox-LDL-treated ECs (Fig. 4d).

### Sirt6-induced autophagy restricted TREM-1-mediated pyroptosis in ox-LDL-treated ECs

**Fig. 5:**
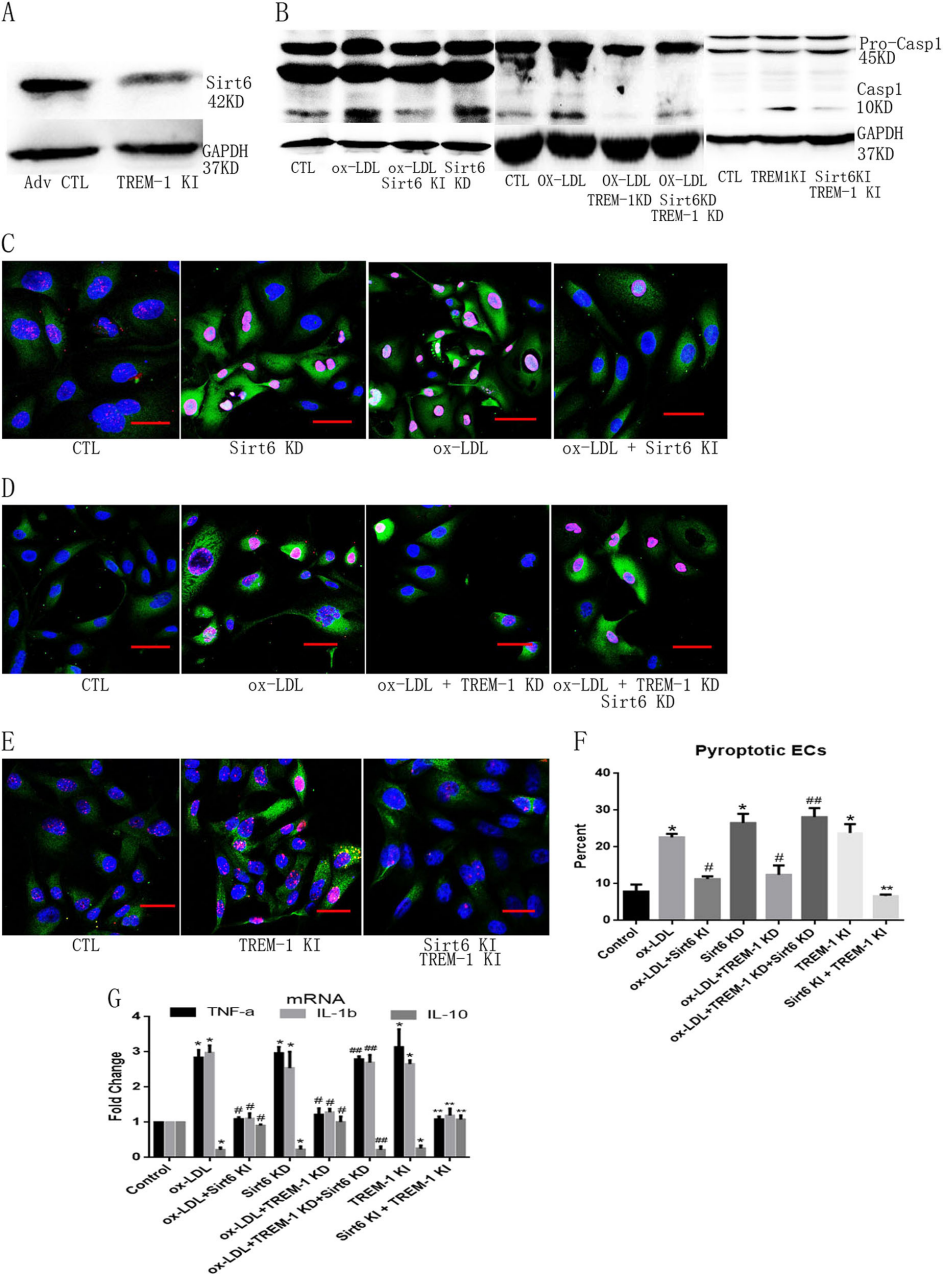
Sirt6 participates in TREM-1-mediated ECs pyroptosis. **a** TREM-1 inhibited Sirt6 expression. **b** TREM-1 and Sirt6 regulated Casp1 maturation. TREM-1 KI or Sirt6 KD directly induced Casp1 maturation, whereas Sirt6 KI effectively abolished ox-LDL- or TREM-1 KI-mediated Casp1 maturation; moreover, TREM-1 KD abrogated ox-LDL-mediated Casp1 maturation in Sirt6 intact ECs, but not in Sirt6 KD ECs. **c** Sirt6 KD simulated ox-LDL-induced pyroptotic cells formation, whereas Sirt6 KI prevented ox-LDL-induced pyroptotic cells formation. Scale bar: 50 μm. **d** TREM-1 KD abrogated ox-LDL-induced pyroptotic cells formation in Sirt6 intact ECs, but not in Sirt6 KD ECs. Scale bar: 50 μm. **e** Sirt6 KI stopped TREM-1 KI-induced pyroptotic cells formation. Scale bar: 50 μm. **f** Quantitation of pyroptotic cells using flow cytometry. **g**. TREM-1 and Sirt6 regulated inflammatory factors expression. TREM-1 KI or Sirt6 KD directly induced inflammatory factors expression, whereas Sirt6 KI effectively abolished ox-LDL- or TREM-1 KI-mediated inflammatory factors expression; moreover TREM-1 KD abrogated ox-LDL-mediated inflammatory factors expression in Sirt6 intact ECs, but not in Sirt6 KD ECs. *N* = 3, *p < 0.05 versus control group; ^#^*p* < 0.05 versus ox-LDL group; **p < 0.05 versus TREM-1 KI group; ^##^*p* < 0.05 versus ox-LDL + TREM-1 KD group

To determine the role of Sirt6-induced autophagy in ECs pyroptosis, Sirt6 expression in TREM-1 KI ECs was firstly detected. We found that TREM-1 KI significantly downregulated Sirt6 expression (Fig. 5a). Then, we evaluated the influence of pyroptotic ECs on Sirt6-managed ECs. We found that Sirt6 KI restrained EC pyroptosis following ox-LDL treatment or TREM-1 KI, whereas Sirt6 KD promoted ECs pyroptosis even in TREM-1 KD ECs (Fig. 5b–f). Also, induced expression of Sirt6 significantly decreased TNF-α and IL-1β mRNA expression, but increased IL-10 mRNA expression in TREM-1 KI or ox-LDL-treated ECs (Fig. 5g). The latter experiments indicated that Sirt6 may reduce TREM-1-mediated pyroptosis in ox-LDL-treated ECs.

**Fig. 6:**
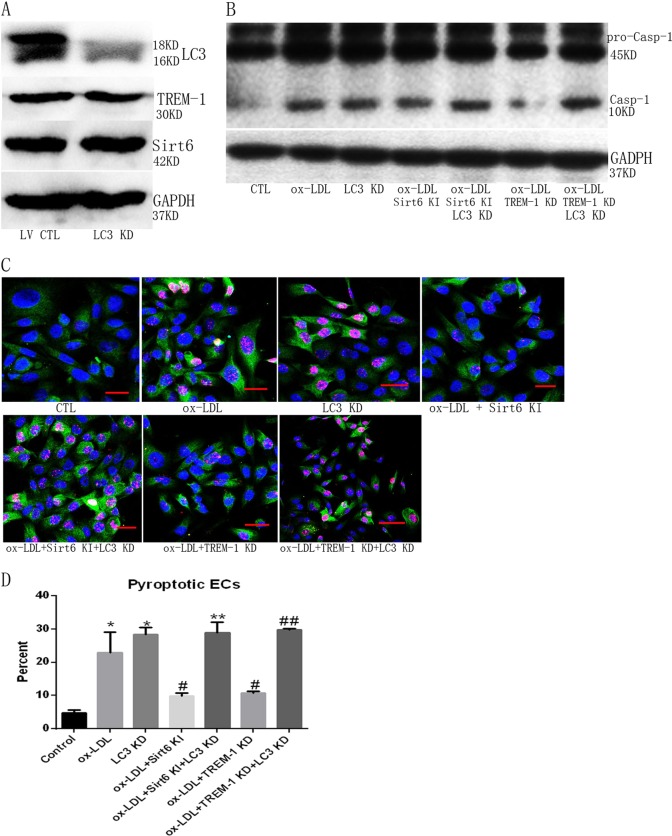
LC3 KD induces pyroptosis. **a** LC3 KD did not change TREM-1 and Sirt6 expression. **b** LC3 KD directly provoked Casp1 maturation in ECs regardless of whether TREM-1 or Sirt6 was intact. **c** LC3 KD directly induced pyroptotic ECs formation regardless of whether TREM-1 or Sirt6 was intact. Scale bar: 50 μm. **d** Quantitation of pyroptotic cells using flow cytometry. *N* = 3, *p < 0.05 versus control group; ^#^*p* < 0.05 versus ox-LDL group; **p < 0.05 versus Sirt6 KI group; ^##^*p* < 0.05 versus ox-LDL + TREM-1 KD group

To further validate that the effect on TREM-1-mediated pyroptosis in ox-LDL-treated ECs is associated with Sirt6-induced autophagy, we established autophagy-deficient ECs using LV-shRNA of LC3 (Fig. 6a). Although LC3 KD did not affect TREM-1 and Sirt6 expression (Fig. 6a), the number of pyroptotic cells was much higher in LC3-deficient ECs (Fig. 6b–d) than LC3 intact ECs.

### sTREM-1, sSirt6, and clinical outcomes

Nine hundred and sixty-two STEMI and NSTEMI patients (median age, 62.7 years; 56.3% men) were enrolled in the study. The level of sTREM-1 was significantly higher in acute myocardial infarction (AMI) patients (median 130.86 pg/ml) than in healthy control (86.38 pg/ml, *n* = 68). In contrast, the levels of sSirt6 were lower in AMI patients (median 312.16 pg/ml) than in healthy controls (859.75 pg/ml, *n* = 68). Patients with higher sTREM-1 (>median) or lower sSirt6 (<median) were elderly and had a history of myocardial infarction and PCI or coronary artery bypass graft (CABG); a history of smoking; nontreatment with ACEI/ARB or statin; and a higher Killip class.

At median 24 months (interquartile range, 18–26) follow-up, the rate of all-cause mortality was 10.19% (*n* = 98), and the rate of the combined major adverse cardiovascular events (MACE) outcome was 18.71% (*n* = 180). Survival analysis showed that after adjustment for variables such as age, male sex, smoking, hypertension, diabetes, hypercholesterolemia, BMI, LVEF, and statin use, the log sTREM-1 was a significant predictor of all-cause mortality (HR: 1.61, 95% CI: 1.15–2.51; *p* < 0.01) and MACE (HR: 2.12, 95% CI: 1.26–3.55; *p* < 0.01), while the log sSirt6 was a negative predictor of all-cause mortality (HR: 0.73, 95% CI: 0.41–0.93; *p* < 0.01) but not of MACE (HR: 0.86, 95% CI: 0.52–1.14; *p* = 0.42).

**Fig. 7:**
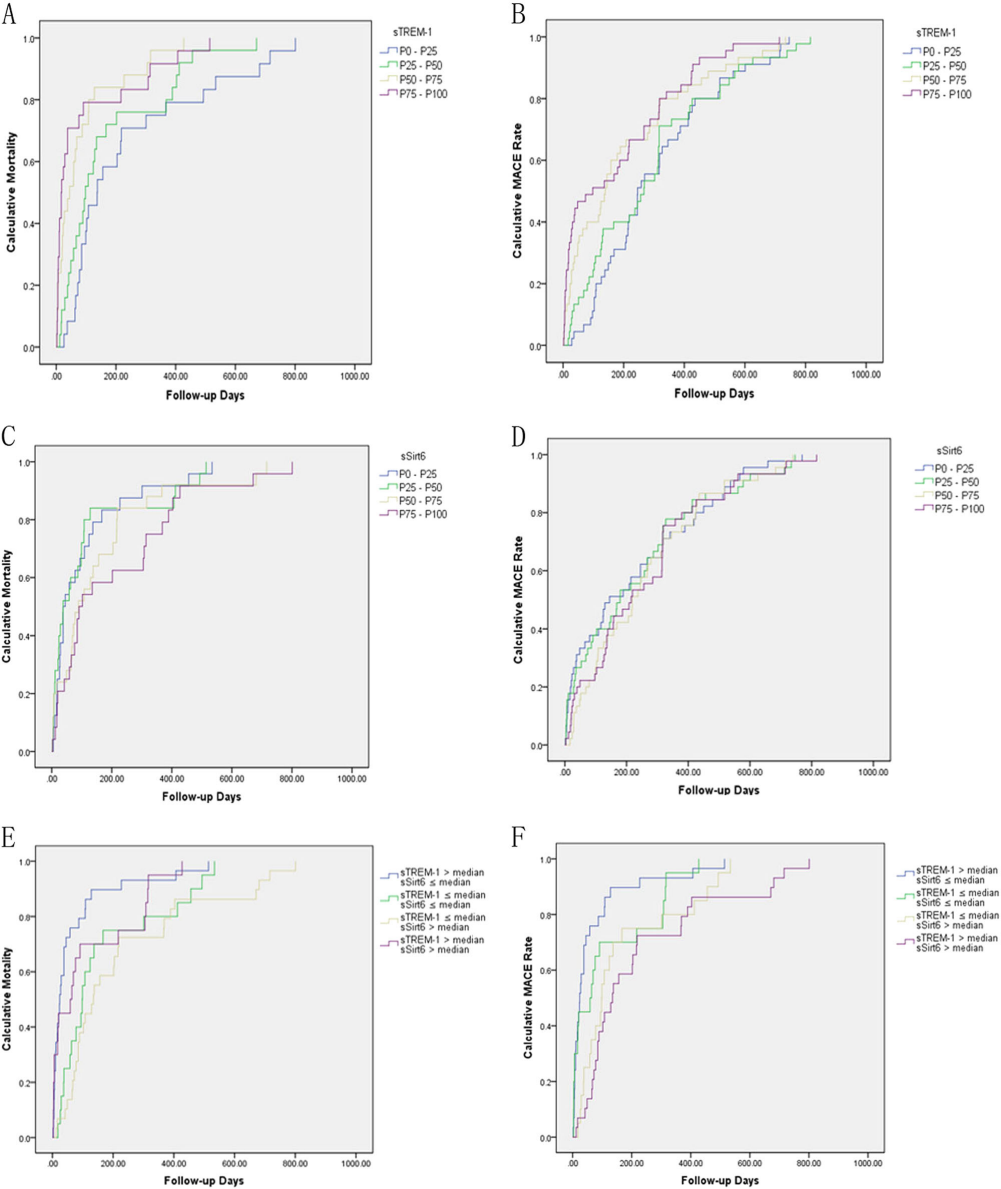
sTREM-1 and sSirt6 levels on admission and 2-year clinical outcomes in 962 AMI patients. High sTREM-1 level was associated with increased risk for all-cause mortality (**a**) and MACE (**b**), while low sSirt6 level was associated with increased risk of all-cause mortality (**c**) but was not associated with MACE (**d**). **e** Patients with high sTREM-1/low sSirt6 had a higher rate of all-cause mortality, whereas those with low sTREM-1/high sSirt6 had a lower rate of all-cause mortality. **f** Patients with high sTREM-1/low sSirt6 had higher rate of MACE, whereas those with low sTREM-1/high sSirt6 had lower rate of MACE

The log-rank test based on the Kaplan–Meier curves also showed a significant association between high sTREM-1 and all-cause mortality (Fig. 7a) and MACE (Fig. 7b), and between low sSirt6 and all-cause mortality (Fig. 7c) but not MACE (Fig. 7d).

We then divided patients into four categories according to high/low sTREM-1 and high/low sSirt6 using a cutoff at the median for sTREM-1 and sSirt6. The results based on the Kaplan–Meier curves showed that patients with high sTREM-1/low sSirt6 had the highest rate of all-cause mortality (Fig. 7e) and MACE (Fig. 7f), whereas the patients with low sTREM-1/high sSirt6 had the lowest risk.

### sTREM-1, sSirt6, and EMPs

In this study, the level of EMPs was significantly higher in AMI patients (median 20,875/ml) than in healthy controls (median 10,218/ml); also the level of EMPs was much higher in patients with all-cause mortality (median 29,000/µl) or MACE (median 24,615/µl).

We also matched the levels of EMP and of sTREM-1/sSirt6. We found that sTREM-1 was related with EMP increase, whereas sSirt6 was related with EMP decrease. By using patients with low sTREM-1/high sSirt6 as the reference group, EMP increase was significantly greater (HR: 6.08, 95% CI: 3.22–10.23; *p* < 0.01). In high sTREM-1/low sSirt6 group, the level of EMPs reached 39000/µl, whereas the level of EMPs was 15,873/µl in low sTREM-1/high sSirt6 group. Additionally, the levels of EMPs were statistically similar in low sTREM-1/low sSirt6 group (20,615/µl) and high sTREM-1/high sSirt6 group (18,598/µl).

## Discussion

We conducted a series of in vitro experiments and in vivo assessments that led to the following findings: In ox-LDL-treated ECs, Sirt6-induced autophagy restricted TREM-1-mediated pyroptosis, and in AMI patients, TREM-1 and in a reversed trend Sirt6 appeared to be markers of endothelial inflammation with potential for use in risk stratification.

ECs are the first cells of the vascular wall to be damaged in atherosclerosis triggered by ox-LDL, glucose, nicotine, and hypertension, and dysfunctional ECs play an important role in the development of atherosclerosis and ultimately in inducing plaque rupture^3^. EC inflammation and subsequent pyroptosis is considered the initiation and critical step in this pathological process^2^. Pyroptosis is a form of regulated cell death triggered by innate immunity, which manifests with specific morphological and molecular features^13^. A peculiar form of chromatin condensation without cellular swelling culminating as well as in plasma membrane permeabilization, and IL-1 and IL-18 release are the two main features of pyroptosis. Initially, pyroptosis was only described as canonical Casp1 activation in monocytes or macrophages^19^. However recent findings indicate that pyroptosis also can be triggered by several other caspases, such as Casp3, Casp11^20^ and is observed in other cell types, such as ECs and alveolar cells^21^. Nevertheless, Casp11- or Casp1-dependent pyroptosis patterns are widely accepted and recognized. Casp11 has a highly specific physical binding domain of LPS, resulting in caspase oligomerization and consequent activation. However, in some cell types including DCs, Casp11 activation is observed in the absence of cell death^22^. Unlike Casp11, Casp1 does not have a canonical LPS binding domain, but it can stimulate inflammasomes formation in microbe-associated molecular patterns in infectious or noninfectious diseases^23^. Casp1-dependent pyroptosis plays key roles in limiting the spread of inflammation, *Nlrc4*^−/−^ mice (which are unable to normally activate Casp1) succumb to low amounts of otherwise innocuous environmental opportunists^24^. In atherosclerotic processing, Casp1-dependent pyroptosis activation seems to play a more critical role, such as in nicotine-mediated ECs dysfunction^9^ and ox-LDL-induced form cell formation^10^. The data presented here show that ox-LDL induces Casp1-dependent pyroptosis activation in ECs; however, Casp11 expression is still nearly undetectable following ox-LDL treatment (data not shown). Therefore, interventions focused on inflammation and how to control phenotype switching of ECs in atherosclerosis might effectively prevent the onset or progression of the disease; however, the underlying mechanism remains unclear.

TREM-1 belongs to the immunoglobulin superfamily and it is believed to play key roles in acute and chronic inflammatory disease. Inhibition of TREM-1 using pharmacological or genetic strategies significantly limits the histological alterations and over-activation of host immune inflammatory responses in experimental models^24^. Of note, recent studies showed that TREM-1 may be a reliable inflammatory mediator in atherosclerotic progression. Studies demonstrate that TREM-1 contributes to promoting monocytosis, monocyte/macrophage proinflammatory responses^25^, VSMCs inflammation, proliferation and migration^15^, and formation of inflammatory foam cells^15^. In clinical studies, sTREM-1 concentration is associated with diabetes^26^ and obesity^27^, which are risk factors for atherosclerosis. In long-term follow-up shows that sTREM-1 concentration is not only associated with increased MACE and mortality in AMI patients^4^ but also associated with in-stent restenosis in ACS patients on statins^16^. Additionally, genetic analysis revealed that TREM-1 gene polymorphisms are closely associated with atherosclerosis severity in a Russian population^28^. Based on the latter evidence, one can posit that elevated TREM-1 might, at least partially, reflect endothelial inflammation in patients with atherosclerosis. Therefore, we assessed sTREM-1 and EMP levels in AMI patients, we found them to be significantly higher, especially in the patients who suffered death or MACE, than in healthy controls. Interestingly, the increase in sTREM-1 was consistent with EMP level. However, previous immunofluorescence data showed that expression of TREM-1 in CD45+ T or leukocytes sporadically localized within the injured artery^16,29^; therefore, the possibility that EC inflammation is secondary to the activation of TREM-1 and downstream pyroptosis cannot be excluded. Fortunately, we found that ox-LDL increased TREM-1 and Casp1 expression, and the proportion of pyroptotic cells among ECs. Direct induction of TREM-1-promoted expression of inflammatory factors, Casp1 activation and EC pyroptosis, which were inhibited by TREM-1 shRNA. Thus, activation of TREM-1 on ECs seems to act as an independent mechanism in promoting inflammatory cell death without other inflammatory cells.

Consistent with previous observations, TREM-1 expression and activity are closely linked with both the NOD-like receptors and Toll-like receptors [TLRs]^30^, which are involved in autophagy, a highly conserved multistep process aimed at maintaining cellular homeostasis, including double membrane structure elongation (marked as p-AMPK, Atg5, and BECN1), autophagosome formation (marked as LC3-II/LC3-I), fusion with lysosome (marked as LAMP2), and finally cargo degeneration (marked as p62)^13^. Meanwhile, TLR activation induces TREM-1 expression in a MyD88-dependent manner^31^. Therefore, TREM-1 is suspected to regulate autophagy as suggested by previous studies. For example, TREM-like transcript-1-derived peptide [LR12] dampens TREM-1 signaling, and TREM-1 genetic KO increases autophagic biomarkers, including TG1/ULK-1, Atg13, Atg5, Atg16L1, LC3-I/II, HSPA8, and HSP90AA1 in colitic mice. Further studies suggested that this effect may be related with TREM-1-mediated mTOR downregulation and AMPK phosphorylation^32^. Interestingly, TLR4 KO may attenuate paraquat-elicited increase in LC3-II/LC3-I, and phosphorylation of AMPK while decreasing phosphorylation of mTOR in cardiomyocyte^33^; the effect of TREM-1 on autophagy thus remains controversial. In our present study, we found that ox-LDL induced TREM-1 activation while impeding autophagy efflux in ECs. Induced expression of TREM-1 directly inhibited autophagy efflux, including blunted autophagosome formation, decreased LC3-II/LC3-I, BECN1, LAMP2 expression, and increased p62 expression. TREM-1 genetic KO using shRNA attenuates ox-LDL-elicited autophagy in ECs. TREM-1 thus might mediate autophagic abrogation in ox-LDL-treated ECs.

Although autophagy is believed to be inhibited in inflammatory disease and the activation of autophagy may restrict inflammation in pathological or physiopathological progression, the accurate role of autophagy in pyroptosis is still controversial. For example, Atg7 deficiency intensifies inflammation and pyroptosis in *Pseudomonas* sepsis^34^ and INF-mediated endometrial epithelial cells^35^. In contrast, autophagy deficiency using 3-MA in benzoapyrene-induced HL-7702 cells may effectively dampen pyroptosis^36^. Interestingly, autophagy restricts pyroptosis in cardiovascular diseases. For instance, autophagy deficiency using 3-MA in acrolein-induced Human umbilical vein endothelial cell (HUVECs) may reinforce pyroptosis, whereas, activating autophagy using rapamycin could inhibit pyroptosis^37^. Much widely used cardiovascular drugs, such as Carvedilol, an α-, β-blocker used to treat congestive heart failure and hypertension, are being tested to attenuate macrophage pyroptosis and induce autophagy activation in a Sirt6 dependent manner^38^. In this study, we demonstrated that direct LC3 interference using shRNA reinforced Casp1 maturation and increased pyroptotic cell proportion. These data underscore that autophagy restricts pyroptosis in many pathological conditions. It is worth noting that most previous studies focused on the role of NLRP3 and pro-IL-1β, the major trigger of pyroptosis, on autophagy. They confirmed that NLRP3 activation may suppress autophagy promotion^35–37^, and autophagy may control pyroptosis progression through decrease IL-1β maturation from pro-IL-1β and export^11^. In our present study, TREM-1 KI directly induced an increase in pyroptotic cell proportion and Casp1 maturation, but inhibited autophagic biomarkers expression and autophagosomes formation, whileTREM-1 KD dampened pyroptosis but restored autophagy in ox-LDL-treated HUVECs. Therefore, TREM-1 may also be a key regulator controlling the pyroptosis/autophagy phenotype transition in ox-LDL-treated HUVECs. However, further molecular studies are needed to gain insight into the mechanisms underlying the control by TREM-1 of the pyroptosis/autophagy phenotype transition.

Recently, several studies documented that cellular pyroptosis/autophagy phenotype transition takes place in a ROS-dependent manner^37^. As a major scavenger of endogenous ROS, Sirtuins are associated with anti-inflammation and pro-autophagy^39^. Sirt6 is one of seven mammalian sirtuins, which plays an important role in cell protection against various stress conditions in cardiovascular diseases, and it is believed that its therapeutic effect may rely on autophagy initiation^17^. Of note, Sirt6 deficiency triggers foam cell formation, endothelial senescence and apoptosis, autophagy inhibition, and plaque progression in atherosclerosis with or without diabetes^40^. Cells overexpressing Sirt6 are characterized by elevated autophagosome number, increased levels of ATG proteins, such as ATG5 and LAMP2, and downregulation of the autophagic inhibition pathway, such as p53 and mTORC1^41^. Also, Sirt6 participates in cholesterol metabolism in THP-1 cells^40^. Our data show that Sirt6 significantly alters number of autophagosomes and the expression of autophagic biomarkers, such as LC3-II/LC3-I, p62, and LAMP2, in ox-LDL-treated ECs, which indicates that Sirt6 induces autophagy and regulates cholesterol metabolism in ECs. Sirt6 also protects cells by suppressing the NF-kB and IL-1β signaling pathway^42^ in inflammation, apoptosis and autophagy induction in both animals and humans and a feature of AS diseases^43,44^. Also, IL-1β is the central factor of pyroptosis^7^. Therefore, Sirt6 is suspected to interfere with pyroptosis. Interestingly, we found that ECs overexpressing Sirt6 are characterized by decreased pyroptotic cell numbers, and TNF-α and IL-1β mRNA levels, whereas Sirt6 deficiency augments ox-LDL-mediated inflammation. The latter effects take place in an autophagy-dependent manner. Once autophagy is blocked using LC3 shRNA, Sirt6-mediated decreases of pyroptotic cells number and Casp-1 level are abrogated. Therefore, our identification of Sirt6-induced autophagy as a regulator of pyroptosis in ECs provides a novel example of the multiple targets and functions of Sirt6 in AS. However, Sirt6 involvement in TREM-1-mediated pyroptosis and the underlying mechanisms have not been studied. Recently, Sirt6 was reported to be crucial in TLR4-, a TREM-1 activator^30^, induced inflammation. Sirt6 overexpression downregulates TLR4 and expression of inflammatory factors such as IL-1β, IL-6, and TNF-α, whereas knocking down of Sirt6 activates TLR4 and inflammatory factors expression^45^. In our present study, TREM-1 KI decreased Sirt6 expression, indicating that TREM-1-mediated proinflammatory effect might rely on Sirt6 abrogation. Sirt6 KI terminates TREM-1 KI or ox-LDL-induced Casp1 maturation, pyroptotic cells formation and inflammatory factor secretion. We also used Sirt6 shRNA to produce Sirt6 KD cells. Sirt6 KD directly induced pyroptosis activation regardless of TREM-1 intactness. These data indicate that TREM-1-mediated pyroptosis may be Sirt6 dependent, which contrasts with the conclusion of a previous study^45^. The cause of this difference may stem from differences in cell lines or their handling. The complex association between TREM-1 and Sirt6 warrants further study.

In conclusion, the data presented here show that ox-LDL induced activation of pyroptosis occurs in a TREM-1 dependent manner. Furthermore, TREM-1 negatively regulated the expression and activation of Sirt6. Moreover, the inhibitory effect of Sirt6 on inflammatory responses might be autophagy dependent. Additionally, in patients with AMI, high levels of TREM-1, and low levels of Sirt6 were associated with an increased risk of all-cause mortality and MACE events at 2 years follow-up. The latter findings suggest that TREM-1-mediated pyroptosis might underlie the pro-atherosclerotic effect of ox-LDL, which may be restricted by Sirt6-induced autophagy in ECs, thereby advancing our understanding of the pathophysiology of ox-LDL-induced ECs dysfunction in atherosclerosis.

## Methods

### Reagents

TRIzol was purchased from Sigma (St Louis, Missouri, USA). TMR red-labeled In-Situ Cell Death Detection reagent and sTREM-1, the sSirt6 enzyme-linked immune sorbent assay (ELISA) kit were purchased from Roche Applied Science (Indianapolis, USA). Monoclonal antibodies against CD62-PE, CD31-PE, CD42-FITC, and IgG-PE were purchased from BD (Shanghai, China), and monoclonal rabbit antibodies against Sirt6, TREM-1, LC3, BECN1, LAMP2, p62, Caspase-1, and GAPDH were purchased from Cell Signaling (Denver, Colorado, USA). Fetal bovine serum (FBS) and Lipofectamine^®^ 2000 Transfection Reagent were purchased from Invitrogen (Carlsbad, CA, USA). The cDNA Synthesis Kit and Premix Ex Taq SYBR Green PCR Kit were purchased from Takara (Shiga, Japan). The *adv-Sirt6, LV-Sirt6 shRNA, adv-TREM-1, LV-TREM-1 shRNA, LV-LC3 shRNA, and GFR, GRP double labeled adv-LC3* were purchased from HanBio. (Shanghai, China). Other unmentioned reagents were purchased from Shenggong Bio. (Shanghai, China).

### Cell culture

HUVEC line was cultured in growth factor supplemented endothelial cell media (ECM) from ScienCell (Carlsbad, California, USA). After incubation with non-FBS ECM for 6 h, HUVECs were reincubated in ECM with 5% FBS and LDL (100 ug/ml), ox-LDL (100 ug/ml) or LPS (1 ug/ml) for 24 h.

### Adv and LV transfection

Adv-control, LV-control, Adv-TREM-1, LV-TREM-1, Adv-Sirt6, LV-Sirt6, or LV-LC3B was directly added to cultured cells and incubated for 48 h.

### Analysis of cell pyroptosis

Two-color flow cytometry and three-color confocal imaging were used to detect cell pyroptosis. HUVECs were incubated with Alexa Fluor 488 labeled caspase-1 at 4 °C overnight, then stained with TMR red-labeled In-Situ Cell Death Detection reagent according to manufacturer’s protocol. Staining with DAPI was used for confocal measurement. The caspase-1 and TMR double stained cells were considered as proptotic cells.

### Analysis of cell autophagic efflux

Two-color confocal imaging was used to detect cell autophagic efflux. HUVECs were incubated with GFP labeled Adv-scramble RNA and GRP labeled Adv-LC3 for 48 h. Then the live cells were immediately examined by confocal imaging. The GFP and GRP double stained organelles were considered to be autophagic efflux.

### Real-time reverse transcription PCR (RT-PCR)

HUVECs were subjected to TRIzol to obtain total RNA according to manufacturer’s instruction. Then, the total RNAs were converted to cDNA using Takara reverse transcriptase and amplified using SYBR Premix reagent for RT-PCR on an ABI 7500 system. The primers (Shenggong Bio., China) were as follows: TNF-α Forward: 5’-CCGTCTCCTACCAGACCAAGG-3, Reverse: 5’-CTGGAAGACCCCTCCCAGATAG-3’; IL-1β Forward: 5’-CTGATGGCCCTAAACAGATGAAG-3’, Reverse: 5’-GGTCGGAGATTCGTAGCAGCTGGAT-3’; IL-10 Forward: 5’-CATGCTGCTGGGCCTGAA-3’, Reverse: 5’-CGTCTCCTTGATCTGCTTGATG-3’.

### Western blotting

HUVECs were subjected to protein lysis buffer (Beyotime, Haimen, china) to obtain total protein according to manufacturer’s instruction. Equal amounts of total protein were subjected to 8–12% SDS-PAGE, transferred to PVDF membranes, blocked with 5% nonfat milk, incubated with primary antibodies at 4 °C overnight, and then coated with HRP-conjugated secondary antibodies at room temperature for 1 h according to standardized protocol. Finally, we used enhanced chemiluminescence reagents (Bio-Max, Israel) to visualize the immunoblots reaction.

### Clinical study population

From October 2012 to December 2015, we prospectively enrolled a total of 962 consecutive patients who presented with ST-segment or non-ST-segment elevation AMI to Shanghai Tongji Hospital, Shanghai East Hospital, Tongji University, and Zhongshan Hospital, Fudan University. AMI was diagnosed according to the “Third universal definition of myocardial infarction”^46^. Individuals younger than 18 years and those with known acute or chronic infectious diseases, liver or renal replacement therapy, malignancy, a suspected or known immunocompromised state, ongoing use or other systemic anti-inflammatory treatments and surgery in the previous 1 month were excluded. Sixty-eight subjects without any signs of ACS and risk factors of CHD were used as healthy control group. All patients signed an informed consent form and accepted management according to usual practice. Blood samples were obtained immediately at the time of admission.

### Clinical study outcomes

All patients prospectively accepted to undergo a follow-up at 2 years after presentation via telephone call to the patients or their families and review of their medical records. The data were entered into a central database and verified by an authorized person.

The clinical study outcomes were 2-year rates of all-cause mortality and MACE including cardiovascular mortality and admission due to recurrent AMI, heart failure or unstable angina that led to urgent revascularization. Heart failure was defined as that treated with high-dose cardiac inotropes, diuretics, or intravenous nitrate.

### Blood samples

Blood samples were collected on admission and centrifuged within 30 min to separate plasma which was stored immediately at −80 ℃. The levels of sTREM-1 and sSirt6 were measured using an established ELISA kit according to the guide manual.

### Plasma EMPs collection and quantitative determination

As previously described [5], after phlebotomy, the blood sample was buffered with sodium citrate immediately and centrifuged at 1,500 g for 10 min, and then, the supernatant fluid was centrifuged at 13,000 g for 20 min, and the pellet was resuspended in PBS to obtain EMPs concentration using flow cytometry. Because CD31 and CD62 are biomarkers for ECs, and CD42 for platelets, CD62+/CD42− or CD31+/CD42− were considered to be EMPs in plasma. CD62+ EMPs are considered to be released from activated ECs, whereas CD31+ EMPs are released from damaged ECs, therefore we detected the plasma levels of CD31+/CD42− EMPs.

### Statistical analysis

Continuous variables are presented as median and interquartile range and were compared by Mann–Whitney U test. Categorical variables are presented as frequencies and were compared by chi-square test or Fisher’s Exact test. Log-rank tests and Cox proportional models were used to analyze the prognostic variables and clinical outcomes during follow-up and expressed as hazard ratios (HRs) with 95% confidence intervals. The factors entered into the Cox proportional models were age, sex, hypertension, diabetes, smoking, hypercholesteremia, body mass index, MI history, PCI or CABG history, AMI type, Killip class, peak troponin I, sTREM-1, sSirt6, and EMP level. The C-statistic was used to evaluate the values of sTREM-1, sSirt6, and EMP in the prediction of all-cause mortality and MACE events. Two-tailed unpaired Student’s t-test or one-way analysis of variance tests were used to determine the significance of the differences among the cellular experimental data. Values of *p* < 0.05 were considered statistically significant. All data analyses were performed using SPSS 20.0 (SPSS Inc., Chicago, USA).
